# Validated semi-supervised early and accurate screening for anterior segment diseases: a 3PM-guided conceptual and technological innovation

**DOI:** 10.1007/s13167-025-00434-3

**Published:** 2026-01-10

**Authors:** Mingyu Xu, Renshu Gu, Zhanyun Lu, Huimin Cheng, Yifan Zhou, Pengjie Chen, Yiming Sun, Jing Cao, Zhichu Chen, Gangyong Jia, Peifang Xu, Juan Ye

**Affiliations:** 1 Zhejiang University, Eye Center of Second Affiliated Hospital, School of Medicine. Zhejiang Provincial Key Laboratory of Ophthalmology. Zhejiang Provincial Clinical Research Center for Eye Diseases. Zhejiang Provincial Engineering Institute on Eye Diseases, Hangzhou, Zhejiang China; 2 Hangzhou Dianzi University, Hangzhou, Zhejiang China

**Keywords:** Predictive preventive personalized medicine (PPPM / 3PM), Preventing irreversible vision loss, Individualized risk stratification, Patient-centered holistic approach, Advanced ophthalmic care, Complex real-word settings, AI, Automated screening system, Ocular anterior segment disease, Slit-lamp imaging

## Abstract

**Background/aims:**

Ocular anterior segment diseases are major causes of global visual impairment. Early and accurate detection of anterior segment abnormalities is essential to support predictive diagnostics, targeted prevention, and individualized treatments management. Conventional slit-lamp assessments are often limited by human observation and inter-clinician variability, restricting their ability to achieve rapid and large-scale disease screening. To advance anterior segment care within the predictive, preventive, and personalized medicine (PPPM/3PM) framework, this study aimed to develop a comprehensive and validated semi-supervised object detection (SSOD) system for slit-lamp imaging–based screening of multiple anterior segment diseases.

**Methods:**

A total of 7230 slit-lamp images from 3302 patients were retrospectively collected at the Second Affiliated Hospital of Zhejiang University between November 2016 and July 2024. The proposed SSOD integrated a Category Control Embed (CCE) module to mitigate class imbalance and an Out-of-distribution Detection Fusion Classifier (ODDFC) to identify previously unseen lesions. Model performance was quantitatively compared with YOLOv8 and ophthalmologists using quantitative metrics (average precision [AP], recall) and clinical assessments of diagnostic accuracy, lesion comprehensiveness, and localization precision.

**Results:**

The SSOD achieved mAP comparable to YOLOv8 (0.729 vs. 0.725 for single-lesion; 0.538 vs. 0.543 for multi-lesion), but demonstrated substantially higher recall (0.893 vs. 0.656 for single-lesion; 0.679 vs. 0.477 for multi-lesion). In clinical evaluations, SSOD scored 2.430/3 for single-lesion and 1.942/3 for multi-lesion detection, outperforming YOLOv8 and approaching the performance of junior ophthalmologists in multi-lesion cases.

**Conclusion:**

The SSOD framework offers an efficient and scalable solution for anterior segment disease screening, delivering reliable multi-lesion detection with minimal annotation. It supports early recognition of anterior segment lesions, guides targeted interventions to prevent irreversible vision loss, and facilitates patient-centered, individualized management that advances ophthalmic care from reactive assessment to proactive precision treatment, aligning with the principles of 3PM.

## Introduction

Ocular anterior segment diseases represent a substantial global health burden, with cataracts being the leading cause of blindness [1] and corneal diseases contributing to blindness or moderate-to-severe visual impairment in approximately 12 million people [2]. Patients affected by anterior segment diseases represent a high-risk population for irreversible vision loss. Within the framework of predictive, preventive, and personalized medicine (PPPM/3PM), a comprehensive medical concept aimed at achieving more effective population-level screening and precision healthcare [3], early recognition and timely intervention for ocular anterior segment diseases are essential for preventing vision-threatening outcomes. Slit-lamp imaging remains a fundamental, accessible, and cost-effective tool for anterior segment evaluation, particularly valuable in primary care and underserved settings [4]. However, its diagnostic accuracy relies heavily on clinical expertise and subjective interpretation, which limits reproducibility and scalability in large populations.

Recent advances in artificial intelligence (AI) have revolutionized ophthalmic image interpretation, offering new opportunities to implement 3PM strategies in eye health [5, 6]. AI-based approaches have demonstrated the capacity to identify disease-specific biomarker combinations for predicting and preventing vision-threatening complications in diabetic retinopathy and proliferative diabetic retinopathy [7, 8], enable noninvasive eyelid disease screening using facial images [9], and integrate structural, functional, and vascular biomarkers for individualized risk stratification of primary open-angle glaucoma progression [10]. Automated analysis of slit-lamp images allows rapid, accurate, and efficient population screening, facilitating early recognition of abnormalities, guiding secondary preventive interventions, and supporting individualized treatment planning through quantitative and interpretable outputs [11].

An ideal 3PM-based automated screening system for anterior segment diseases should meet several key criteria: it should cover common anterior segment disorders, be applicable to complex clinical scenarios including both single-disease and multi-disease cases, provide accurate and reliable predictions, and offer interpretable lesion information to assist clinicians in personalized decision-making. However, most existing AI models are limited to single-disease detection and fail to capture the frequent coexistence of multiple anterior segment disorders [12–15]. This limitation constrains predictive capacity and clinical applicability in complex real-world settings. Our previous work addressed this issue through a multi-label, multi-disease classification system for 13 common ocular conditions [16]. However, similar to other frameworks [17–19], the absence of interpretable lesion localization hindered clinical transparency and practical adoption. To achieve robust predictive performance, several technical challenges must be addressed. First, large-scale annotated datasets require substantial expert effort, restricting the scalability of model training [20, 21]. Second, class imbalance within real-world datasets introduces systematic bias, compromising the predictive validity of rare but clinically significant lesions [22]. Third, the open-set nature of clinical imaging necessitates algorithms capable of recognizing previously unseen or atypical lesion types, which is crucial for extending predictive capability to complex clinical scenarios [23]. These challenges underscore the urgent need for an AI framework that not only achieves high diagnostic accuracy, but also provides interpretable lesion information, maintains robustness across diverse clinical scenarios, and supports personalized decision-making.

## Working hypothesis and purpose in the framework of 3PM

Current clinical management of anterior segment diseases faces major challenges. Many ocular surface abnormalities, such as cataract- and cornea-related lesions, progress silently in their early stages and are often detected only after irreversible complications occur, resulting in delayed intervention and increased blindness risk. In addition, the frequent coexistence of multiple anterior segment disorders complicates diagnosis and treatment, underscoring the urgent need for a comprehensive, accurate, and interpretable screening tool that can capture complex, multifactorial pathology.

In alignment with the principles advocated by the European Association for Predictive, Preventive, and Personalized Medicine (EPMA) [3], we developed a SSOD framework for comprehensive slit-lamp–based screening of anterior segment disorders. The system was designed to achieve wide disease coverage by encompassing 12 common anterior segment pathologies and their coexisting lesions, thereby improving diagnostic completeness and clinical practicality. Importantly, the framework provides interpretable outputs by classifying each detected lesion and precisely localizing it within the anterior segment, offering clinicians actionable and visualized information to guide individualized decision-making. To enhance predictive performance while minimizing annotation burden, the semi-supervised architecture leverages limited labeled data effectively, making it scalable for large-scale screening programs. Furthermore, the integration of the CCE module dynamically mitigates class imbalance, while the ODDFC identifies novel or atypical lesion patterns, extending the system’s capability beyond predefined disease categories.

From a predictive perspective, the framework enhances the sensitivity to early lesions, helping clinicians identify individuals with vison-threatening anterior segment diseases before irreversible vision loss occurs. From a preventive perspective, it supports rapid, efficient, and cost-effective large-scale screening, assisting in the implementation of timely, targeted interventions that can halt disease progression. From a personalized perspective, the interpretable multi-lesion localization output provides fine-grained visualization of pathological areas, enabling clinicians to assess lesion severity and tailor individualized treatment strategies. This approach represents a practical realization of the 3PM paradigm—providing a robust and efficient solution to advance proactive, precision-oriented ophthalmic care, particularly in primary and resource-limited settings [24].

## Methods

### Datasets

**Fig. 1:**
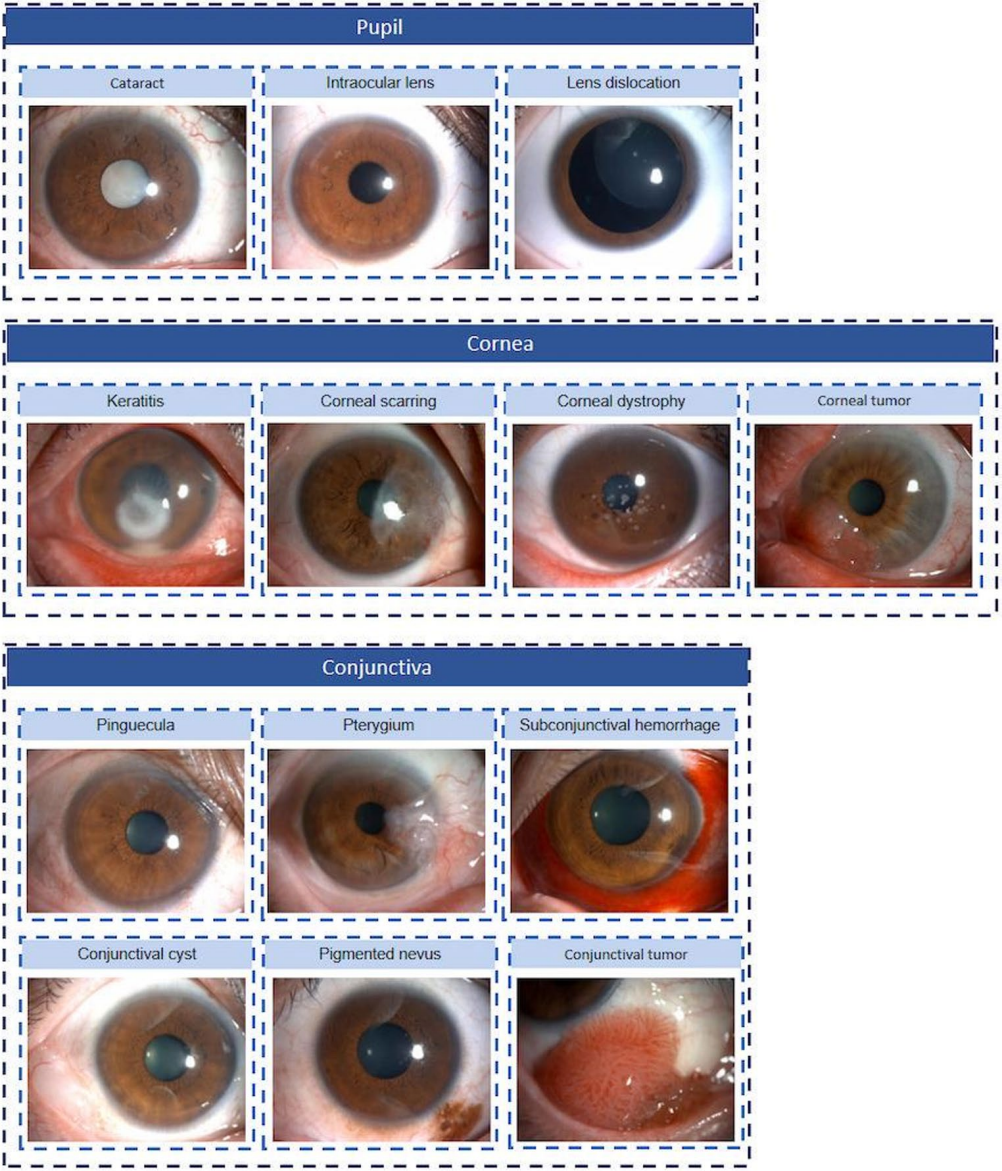
Example images of 12 ocular anterior segment diseases

The study employed a comprehensive dataset of anterior segment slit-lamp images encompassing 12 common ocular conditions, systematically categorized according to standard ophthalmological references including EyeWiki [25], Wills Eye Manual [26], and Digital Reference of Ophthalmology [27]. These conditions were further grouped by anatomical region: (1) pupillary zone pathologies, comprising cataract, intraocular lens, and lens dislocation; (2) corneal disorders, including keratitis, corneal scarring, corneal dystrophy, and corneal tumors; and (3) conjunctival abnormalities, consisting of pinguecula, pterygium, subconjunctival hemorrhage, conjunctival cyst, pigmented nevus, and conjunctival tumors. Because tumorous lesions may involve both corneal and conjunctival tissues, they were consolidated into a unified category of corneal/conjunctival tumor. Representative images of the 12 conditions are provided in Fig. 1.

All images were acquired at the Second Affiliated Hospital of Zhejiang University using standardized Topcon SL-D701 slit-lamp biomicroscopes equipped with DC-4 digital cameras between November 2016 and July 2024. Inclusion criteria required images with adequate clarity to allow definitive lesion identification and visualization of at least one major anterior segment region. Exclusion criteria included images containing non-target pathologies, slit-beam illumination, or cobalt blue light.

### Labeling

A dedicated annotation team was established to ensure high-quality and consistent labeling of all slit-lamp images. The team consisted of one junior ophthalmologist (JO) with over 3 years of clinical experience, one senior ophthalmologist (SO) with more than 5 years of experience, and one specialized ophthalmologist with more than 10 years of expertise. The JO and SO independently annotated the images using the VGG Image Annotator tool, and all annotations were subsequently reviewed and confirmed by the specialized ophthalmologist to resolve discrepancies. For each of the 12 target lesions, bounding boxes were applied to delineate lesion boundaries, accompanied by categorical labels specifying lesion type.

### Development of the SSOD system

**Fig. 2:**
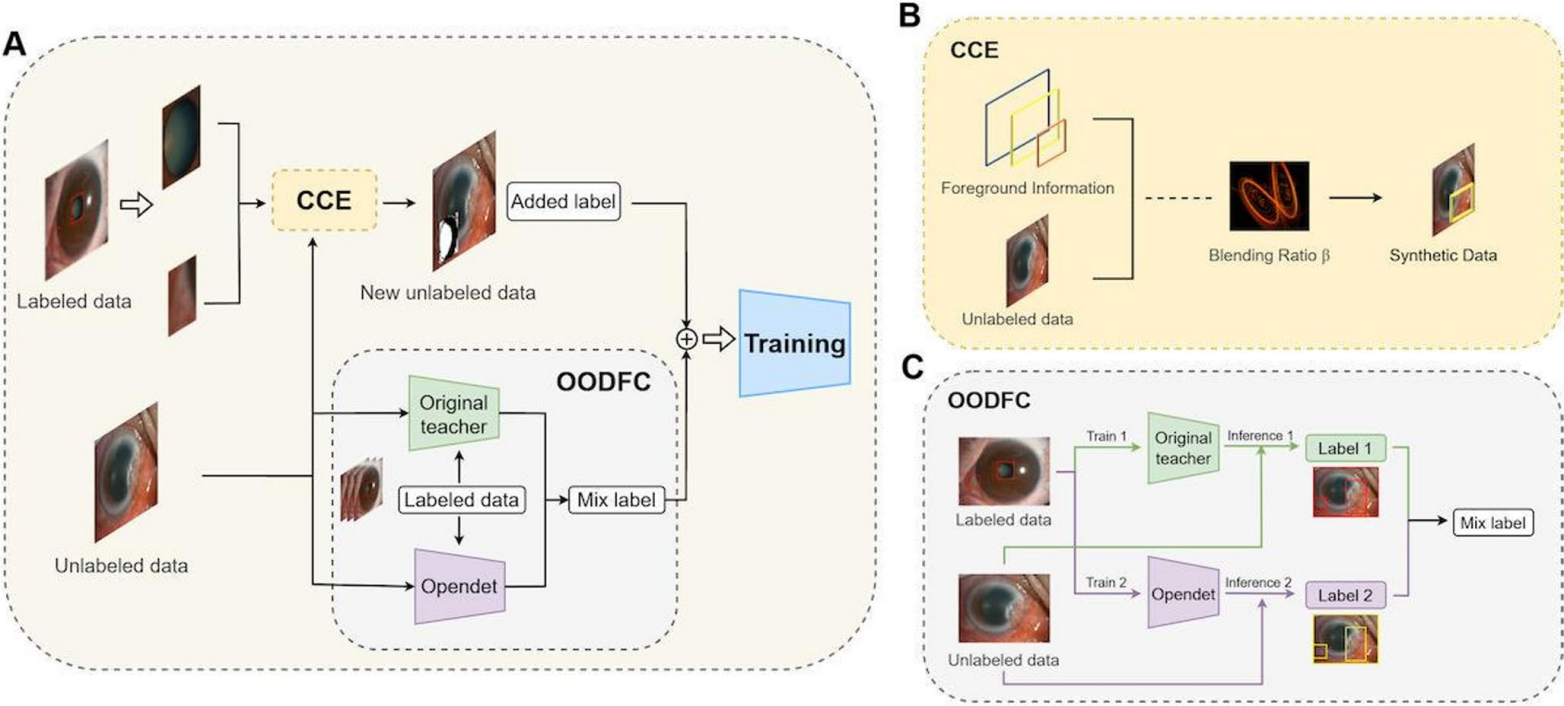
The network architecture of the SSOD system. (**a**) the overall structure of the SSOD algorithm, (**b**) the Category Control Embed (CCE) module, (**c**) the Out-of-distribution Detection Fusion Classifier (OODFC) module

As illustrated in Fig. 2a and previously described [28], our screening framework builds upon the Mean-Teacher semi-supervised object detection (SSOD) paradigm, enhanced with two specialized modules designed to address key challenges in medical image analysis. The overall training objective combines supervised and unsupervised components:

$$\:L={L}_{s}+\lambda\:{L}_{u}$$

where $\:{\uplambda\:}$ balances the contributions of the supervised and unsupervised losses. The supervised loss is defined as

$$\:{L}_{s}={L}_{cls}+{L}_{loc}$$.

while the unsupervised loss incorporates consistency regularization with refined pseudo-labeling:

$$\:{L}_{\mathrm{consistency}}={\sum\:}_{i=1}^{N}\left|\right|{f}_{{\uptheta\:}}\left({x}_{i}\right)-{f}_{{\uptheta\:}}\left(T\left({x}_{i}\right)\right)|{|}^{2}$$

where $\:T\left({x}_{i}\right)$ represents a transformed version of $\:{x}_{i}$, and $\:{f}_{{\uptheta\:}}\:$denotes the model’s prediction function.

The first module, Category Control Embed (CCE), mitigates class imbalance by constructing a dynamic Foreground Information Library that selectively oversamples rare categories while undersampling dominant ones. Foreground segments extracted from labeled data are blended with unlabeled images to generate balanced synthetic training samples, formulated as:

$$\:{x}_{i}^{\mathrm{S}\mathrm{yn}}=\beta\:{b}_{j}^{{\prime\:}}+\left(1-\beta\:\right){x}_{i},\:{\:{y}_{i}}^{\mathrm{Syn}}=\mathrm{combine}\left({y}_{i},{y}_{j}\right)={y}_{j},$$

where $\:{b}_{j}^{{\prime\:}}$ is an augmented foreground segment, and $\:{x}_{i}$ is a randomly selected region from an unlabeled image fused with $\:{b}_{j}^{{\prime\:}}$, ensuring equitable class representation during model optimization (Fig. 2b).

The second module, Out-of-Distribution Detection Fusion Classifier (OODFC), tackles the open-set problem by introducing an auxiliary detector to identify previously unseen categories within unlabeled data. Its predictions are fused with the teacher model outputs using a category-specific adaptive threshold:

$$\:{T}_{i}=\mathrm{max}\left(0,\mathrm{min}\left(1,{e}^{{\upgamma\:}\cdot\:\left(A{P}_{i}-1\right)}\right)\right)$$

where $\:{T}_{i}$ determines whether to retain the label of an unknown category, $\:A{P}_{i}$ is the Average Precision of category.

𝑖 from the fully supervised teacher network, and $\:\gamma\:$ controls the growth rate of the exponential function. This design reduces misclassification of novel lesions while maintaining high accuracy for known categories (Fig. 2c).

Both modules are seamlessly integrated into a Mean-Teacher [29–32] pipeline that employs ResNet-50 [33] as the backbone, Feature Pyramid Network (FPN) [34] as the neck, and Fully Convolutional One-Stage Object Detector (FCOS) [35] as the detection head. Within the teacher–student paradigm, the teacher model is updated via Exponential Moving Average (EMA),

$$\:{{\uptheta\:}}_{t+1}={\upalpha\:}{{\uptheta\:}}_{t}+\left(1-{\upalpha\:}\right){{\uptheta\:}}_{t+1}^{{\prime\:}}$$

where $\:\alpha\:$ is a decay coefficient and $\:{\theta\:}_{t+1}^{{\prime\:}}$ denotes the current model parameters, ensuring stable evolution and high-quality pseudo-label generation from both original and CCE-augmented data. By unifying CCE and OODFC within the SSOD framework, the proposed system effectively addresses the dual challenges of class imbalance and open-set recognition while maintaining computational efficiency, thereby achieving superior performance in semi-supervised detection of ocular anterior segment lesions.

In our experiments, a rigorous data partitioning scheme was implemented. The training set included 31% of single-lesion images spanning 12 pathological categories, 50% of unknown-class samples, and all unlabeled images, while the validation set comprised the remaining 69% of single-lesion images, the other 50% of unknown-class samples, and all multi-lesion cases. Single-lesion images facilitated focused learning of core pathological features, whereas multi-lesion images were reserved exclusively for validation to evaluate performance on complex, clinically representative scenarios. The partial inclusion of unknown-class data in training enabled controlled adaptation to open-set conditions. This partitioning strategy ensured comprehensive exposure to fundamental lesion patterns while maintaining a realistic open-set evaluation environment. Detailed data distribution, including sample counts per category and split, is provided in Table 1.

**Table 1:** Statistics of the slit-lamp image dataset

Classification		No. of slit-lamp images		
		Training set	Validation set	Total
Single-lesion	Cataract	34	73	107
	Intraocular lens	22	47	69
	Lens dislocation	10	21	31
	Keratitis	51	111	162
	Corneal scarring	16	33	49
	Corneal dystrophy	90	199	289
	Corneal/conjunctival tumor	139	308	447
	Pinguecula	63	140	203
	Pterygium	29	63	92
	Subconjunctival hemorrhage	42	92	134
	Conjunctival cyst	28	62	90
	Pigmented nevus	119	263	382
Multi-lesion		0	175	175
Unlabeled images		4500	0	4500
Unknown type	Ocular trauma	250	250	500

### Evaluation of the lesion detection ability of the SSOD system

We implemented a comprehensive dual evaluation framework combining quantitative metrics and clinical assessment to rigorously evaluate model performance. Quantitative evaluation employed recall and Average Precision (AP) metrics following standard object detection protocols with the MMDetection object detection benchmark [36]. In our experiments, AP is computed as the exact area under the Precision-Recall curve by numerical integration, then averaged over Intersection over Union (IoU) thresholds, categories, and object-area ranges. Recall was reported as a global average recall. Mean Average Precision (mAP), defined as the mean of AP values across all classes, was used as an overall indicator of model performance across the dataset. In addition, precision, recall, and F1-score were reported at a confidence threshold of 0.3, which was selected to balance sensitivity and specificity in a manner consistent with clinical screening requirements.

Three model configurations were systematically compared: SSOD_1, treating ocular trauma as an unknown class; SSOD_2, treating intraocular lens, corneal dystrophy, and subconjunctival hemorrhage as unknown classes, selected as representative diseases from distinct anatomical regions; and YOLOv8 [37] as a benchmark. Performance was evaluated separately on single-lesion and multi-lesion images, with the latter assessing model capability in complex clinical scenarios.

For qualitative clinical evaluation, the SSOD models were compared with YOLOv8 using three criteria: diagnostic accuracy (scored 1 for correct, 0 for incorrect diagnosis, reflecting misdiagnosis), lesion comprehensiveness (scored 1 for complete detection, 0 for missed lesions, reflecting underdiagnosis), and localization precision (scored 1 for accurate bounding boxes, 0.5 for partially accurate boxes with acceptable positional deviations, and 0 for clearly inaccurate boxes). The composite clinical score, summing these three components, ranged from 0 to 3. Evaluations were performed separately on single-lesion and multi-lesion images.

To establish clinical benchmarks, three junior ophthalmologists and one senior ophthalmologist, independent of the original annotation team, assessed 127 single-lesion images covering all 12 disease categories and 15 randomly selected multi-lesion images from the validation set. This blinded expert evaluation enabled direct comparison between human and model performance under identical assessment criteria.

### Statistical analysis

For quantitative evaluation, AP and recall metrics were computed to assess lesion detection performance of the SSOD and YOLOv8 systems. In addition, to facilitate operating-point–based comparison, precision, recall, and F1-score were further calculated at a confidence threshold of 0.3.

For clinical evaluation, manual scoring of three key diagnostic measures—diagnostic accuracy, lesion comprehensiveness, and localization precision—was performed to compare the performance of SSOD, YOLOv8, and ophthalmologists.

## Results

### Characteristics of the dataset

**Fig. 3:**
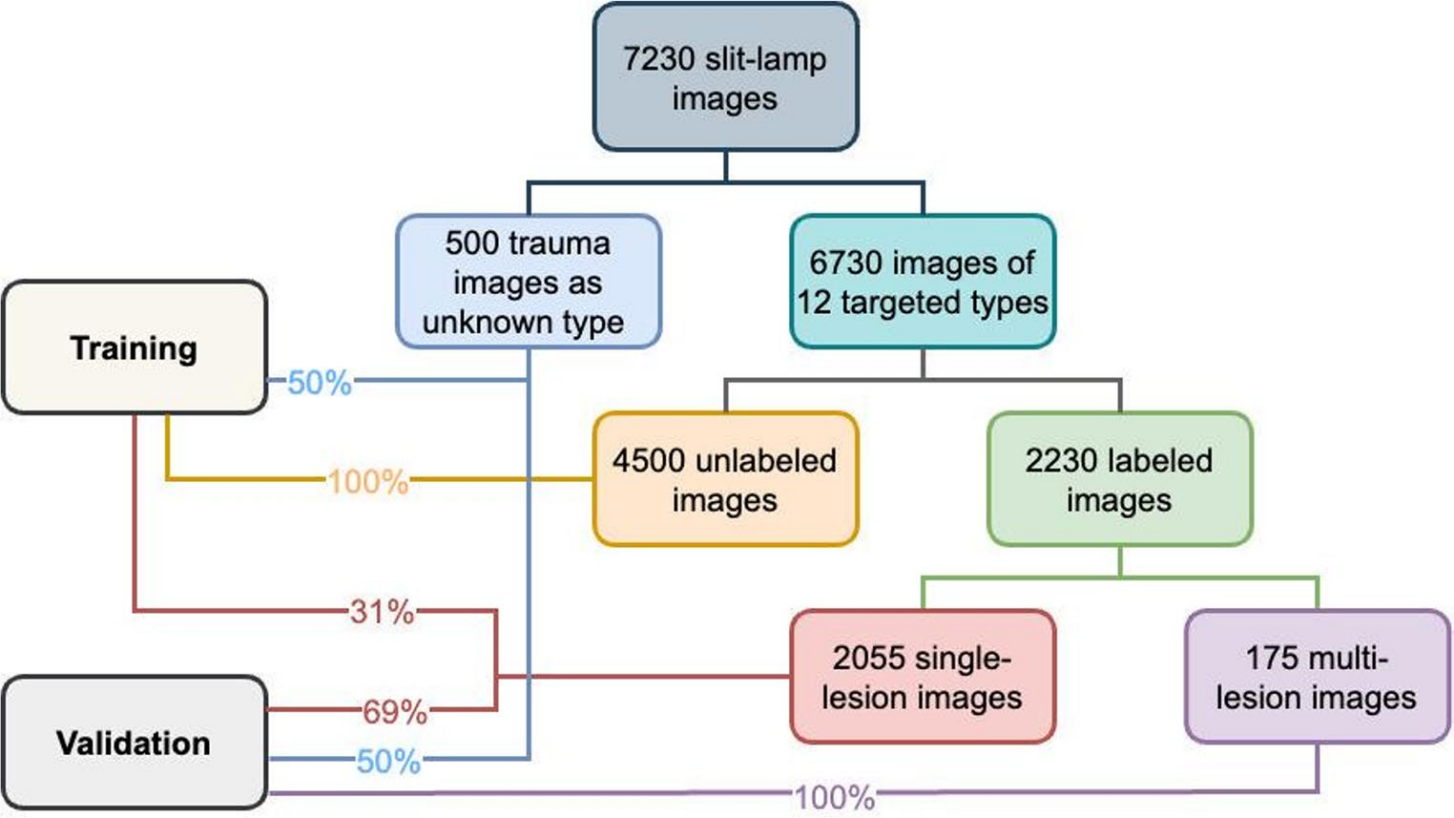
The data distribution statistics of slit-lamp image dataset

The study included a total of 7230 slit-lamp images obtained from 3302 patients between November 2016 and July 2024. As shown in Fig. 3, the dataset comprised two distinct subsets: (1) 6730 images representing the 12 target ocular pathologies, and (2) 500 images of ocular anterior segment trauma, serving as out-of-distribution examples. Among the pathology images, 2230 were annotated by ophthalmologists (2055 single-lesion and 175 multi-lesion images), while the remaining 4500 images were left unlabeled. Detailed image distribution across specific pathology categories is provided in Table 1.

### Quantitative evaluation of the SSOD system

#### Performance on single-lesion images

We evaluated the lesion detection performance of SSOD_1, SSOD_2, and YOLOv8 on single-lesion images using mAP and recall metrics. SSOD_1 treated ocular trauma as an unknown class, whereas SSOD_2 treated intraocular lens, corneal dystrophy, and subconjunctival hemorrhage as unknown classes.

**Fig. 4:**
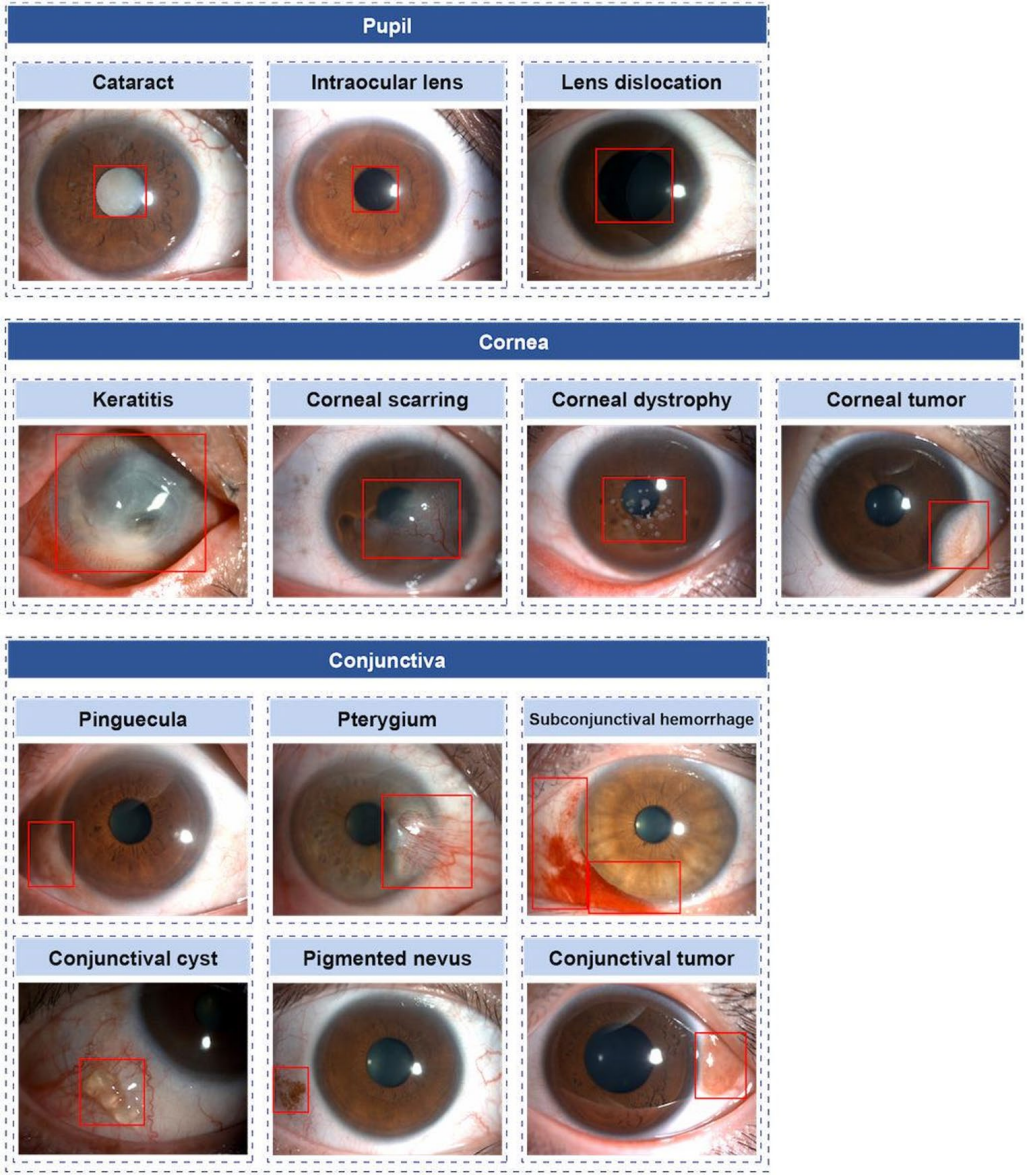
Example images of the prediction results of the SSOD system on 12 ocular anterior segment diseases

As shown in Table 2, SSOD_1 achieved higher overall mAP (0.729 vs. 0.687) with comparable recall (0.893 vs. 0.880) relative to SSOD_2, and detected a greater number of lesion types (12 vs. 9). Compared with YOLOv8 (mAP = 0.725, recall = 0.656), SSOD_1 exhibited markedly higher recall, indicating superior capability to minimize missed diagnoses, which is critical for clinical screening. Predicted bounding boxes for all 12 lesions are presented in Fig. 4.

**Table 2:** The performance of SSOD_1, SSOD_2, and YOLOv8 for detecting 12 ocular anterior segment diseases in single-lesion slit-lamp images

Classification	SSOD_1		SSOD_2		YOLOv8	
	Recall	AP	Recall	AP	Recall	AP
Cataract	1.000	0.887	1.000	0.887	0.877	0.847
Intraocular lens	0.957	0.842	-	-	0.851	0.869
Lens dislocation	1.000	0.665	1.000	0.495	0.744	0.796
Keratitis	0.823	0.652	0.837	0.549	0.617	0.643
Corneal scarring	0.683	0.474	0.585	0.407	0.366	0.488
Corneal dystrophy	0.961	0.814	-	-	0.837	0.925
Corneal/conjunctival tumor	0.923	0.765	0.926	0.770	0.699	0.810
Pinguecula	0.917	0.779	0.903	0.746	0.481	0.591
Pterygium	1.000	0.954	1.000	0.973	0.905	0.942
Subconjunctival hemorrhage	0.826	0.556	-	-	0.430	0.493
Conjunctival cyst	0.747	0.611	0.787	0.599	0.402	0.533
Pigmented nevus	0.879	0.754	0.886	0.755	0.657	0.765

Region-wise analysis demonstrated robust performance across anatomical areas: pupillary zone pathologies (mAP = 0.798, recall = 0.986), corneal lesions (mAP = 0.676, recall = 0.848), and conjunctival abnormalities (mAP = 0.737, recall = 0.882). Lower performance was noted for corneal scars and subconjunctival hemorrhage, likely due to variable morphology and diffuse lesion distribution, respectively. Notably, for clinically critical conditions—including cataract, lens dislocation, keratitis, corneal/conjunctival tumor and pterygium—SSOD_1 demonstrated excellent detection capability, with mAP = 0.785 and recall = 0.949.

In addition, when evaluated at a confidence threshold of 0.3, SSOD_1 further demonstrated favorable operating-point performance in the single-lesion scenario. As shown in Table 3, SSOD_1 achieved the highest F1-score among the three models (0.710 vs. 0.654 for SSOD_2 vs. 0.701 for YOLOv8), reflecting a more balanced trade-off between precision and recall under screening-oriented conditions. These results are consistent with the threshold-agnostic evaluation and further support the robustness of SSOD_1 for single-lesion anterior segment screening.

**Table 3:** Precision, recall, and F1-score of SSOD_1, SSOD_2, and YOLOv8 at a confidence threshold of 0.3 for detecting 12 ocular anterior segment diseases in single-lesion slit-lamp images

Classification	SSOD_1			SSOD_2			YOLOv8		
	Precision	Recall	F1 score	Precision	Recall	F1 score	Precision	Recall	F1 score
Cataract	0.716	0.863	0.783	0.500	0.973	0.661	0.724	0.877	0.793
Intraocular lens	0.784	0.851	0.816	-	-	-	0.670	0.851	0.750
Lens dislocation	0.519	0.667	0.584	0.278	0.714	0.400	0.690	0.744	0.716
Keratitis	0.723	0.610	0.662	0.527	0.617	0.568	0.661	0.617	0.638
Corneal scarring	0.824	0.341	0.482	0.750	0.366	0.492	0.961	0.366	0.530
Corneal dystrophy	0.827	0.823	0.825	-	-	-	0.939	0.841	0.887
Corneal/conjunctival tumor	0.769	0.785	0.777	0.746	0.788	0.766	0.888	0.699	0.782
Pinguecula	0.870	0.648	0.743	0.738	0.738	0.738	0.786	0.482	0.598
Pterygium	0.724	1.000	0.840	0.775	0.984	0.867	0.902	0.905	0.903
Subconjunctival hemorrhage	0.793	0.436	0.563	-	-	-	0.591	0.430	0.498
Conjunctival cyst	0.870	0.533	0.661	0.844	0.507	0.633	0.905	0.400	0.555
Pigmented nevus	0.855	0.730	0.788	0.801	0.730	0.764	0.901	0.654	0.758

#### Performance on multi-lesion images

Given the frequent co-occurrence of multiple ocular pathologies in clinical practice, we further evaluated model performance on multi-lesion images to assess its capability in complex clinical scenarios, with detailed results shown in Table 4. Evaluation of lens dislocation and corneal degeneration was not possible in the 175 multi-lesion images due to their absence.

SSOD_1 and SSOD_2 exhibited similar mAP and recall (mAP: 0.538 vs. 0.544; recall: 0.679 vs. 0.684), but SSOD_1 detected a greater number of lesion types (12 vs. 9), indicating broader applicability. Compared with YOLOv8 (mAP = 0.543, recall = 0.477), SSOD_1 achieved substantially higher recall while maintaining comparable mAP, reflecting an enhanced ability to minimize missed diagnoses in complex multi-lesion cases. Although overall performance on multi-lesion images was lower than on single-lesion images, SSOD_1 maintained adequate detection capability. For four clinically critical conditions present in the multi-lesion set—cataract, keratitis, corneal/conjunctival tumors, and pterygium—SSOD_1 achieved mAP = 0.705 and recall = 0.870, demonstrating robust detection performance for clinically significant pathologies irrespective of lesion complexity.

In addition, when evaluated at a confidence threshold of 0.3, the operating-point–based metrics further highlighted the comparative performance of the models in the multi-lesion scenario. As summarized in Table 5, SSOD_1 achieved a higher F1-score than YOLOv8 (0.521 vs. 0.514). Although SSOD_2 yielded a higher F1-score of 0.547, SSOD_1 detected a markedly broader spectrum of lesion categories (12 vs. 9), indicating greater coverage and potential clinical applicability in complex multi-lesion settings. These findings are consistent with the threshold-agnostic results and support the robustness of SSOD_1 for real-world multi-lesion screening.

**Table 4:** The performance of SSOD_1, SSOD_2, and YOLOv8 for detecting 12 ocular anterior segment diseases in multi-lesion slit-lamp images

Classification	SSOD_1		SSOD_2		YOLOv8	
	Recall	AP	Recall	AP	Recall	AP
Cataract	0.845	0.744	0.958	0.849	0.679	0.795
Intraocular lens	0.964	0.909	-	-	0.893	0.969
Lens dislocation	-	-	-	-	-	-
Keratitis	0.885	0.648	0.846	0.680	0.654	0.643
Corneal scarring	0.286	0.117	0.143	0.015	0.000	0.042
Corneal dystrophy	-	-	-	-	-	-
Corneal/conjunctival tumor	0.818	0.674	0.758	0.661	0.576	0.594
Pinguecula	0.468	0.422	0.621	0.515	0.317	0.546
Pterygium	0.931	0.752	0.931	0.799	0.727	0.807
Subconjunctival hemorrhage	0.448	0.284	-	-	0.284	0.324
Conjunctival cyst	0.643	0.435	0.714	0.456	0.357	0.396
Pigmented nevus	0.500	0.397	0.500	0.373	0.286	0.311

**Table 5:** Precision, recall, and F1-score of SSOD_1, SSOD_2, and YOLOv8 at a confidence threshold of 0.3 for detecting 12 ocular anterior segment diseases in multi-lesion slit-lamp images

Classification	SSOD_1			SSOD_2			YOLOv8		
	Precision	Recall	F1 score	Precision	Recall	F1 score	Precision	Recall	F1 score
Cataract	1.000	0.549	0.709	0.931	0.761	0.837	0.929	0.549	0.690
Intraocular lens	1.000	0.821	0.902	-	-	-	1.000	0.893	0.943
Lens dislocation	-	-	-	-	-	-	-	-	
Keratitis	0.789	0.577	0.667	0.708	0.654	0.680	0.763	0.615	0.681
Corneal scarring	0.000	0.000	0.000	0.000	0.000	0.000	0.000	0.000	0.000
Corneal dystrophy	-	-	-	-	-	-	-	-	-
Corneal/conjunctival tumor	0.900	0.545	0.679	0.875	0.636	0.737	0.745	0.444	0.556
Pinguecula	0.962	0.202	0.334	0.938	0.242	0.385	0.930	0.216	0.351
Pterygium	0.793	0.793	0.793	0.893	0.862	0.877	0.860	0.655	0.744
Subconjunctival hemorrhage	0.769	0.149	0.250	-	-	-	0.476	0.231	0.311
Conjunctival cyst	0.667	0.286	0.400	0.800	0.286	0.421	0.833	0.357	0.500
Pigmented nevus	0.714	0.357	0.476	0.556	0.357	0.435	0.484	0.286	0.360

### Clinical assessment of the SSOD system

Beyond automated quantitative evaluation, we performed clinical assessment to examine the practical screening value of the models using three metrics: diagnostic accuracy, lesion comprehensiveness, and localization precision. Comparative results between SSOD_1 and YOLOv8 for both single-lesion and multi-lesion images are summarized in Table 6.

On single-lesion images, SSOD_1 outperformed YOLOv8 with a total clinical score of 2.430/3 versus 2.366/3, driven primarily by higher diagnostic accuracy (0.916/1 vs. 0.862/1). Notably, for five clinically critical conditions—cataract, lens dislocation, keratitis, corneal/conjunctival tumor, and pterygium—SSOD_1 demonstrated excellent performance with a total score of 2.602, highlighting its robust utility in clinically significant scenarios. In multi-lesion images, SSOD_1 maintained its advantage, achieving a total score of 1.942/3 compared with 1.820/3 for YOLOv8, again reflecting superior diagnostic accuracy (0.954/1 vs. 0.829/1).

**Table 6:** Comparative evaluation of SSOD_1 and YOLOv8 on diagnostic accuracy, comprehensiveness, and location precision

Classification		SSOD_1					YOLOv8				
		Accuracy	Comprehen-siveness	Location	Total		Accuracy	Comprehen-siveness	Location	Total	
Single-lesion	Cataract	1.000	0.836	0.836	2.672		0.932	0.918	0.918	2.768	
	Intraocular lens	0.872	0.830	0.830	2.532		0.809	0.851	0.851	2.511	
	Lens dislocation	0.905	0.810	0.810	2.525		0.762	0.762	0.762	2.286	
	Keratitis	0.874	0.685	0.680	2.239		0.847	0.757	0.716	2.320	
	Corneal scarring	0.818	0.424	0.424	1.666		0.636	0.455	0.439	1.530	
	Corneal dystrophy	0.975	0.874	0.869	2.718		0.965	0.915	0.910	2.790	
	Corneal/conjunctival tumor	0.938	0.838	0.830	2.606		0.899	0.828	0.794	2.521	
	Pinguecula	0.900	0.750	0.746	2.396		0.857	0.636	0.618	2.111	
	Pterygium	1.000	0.984	0.984	2.968		0.921	0.905	0.905	2.731	
	Subconjunctival hemorrhage	0.902	0.696	0.674	2.272		0.879	0.769	0.665	2.313	
	Conjunctival cyst	0.887	0.548	0.524	1.959		0.887	0.548	0.524	1.959	
	Pigmented nevus	0.924	0.856	0.829	2.609		0.954	0.821	0.771	2.546	
Multi-lesion		0.954	0.496	0.492	1.942		0.829	0.503	0.488	1.820	

### Comparison with human ophthalmologists

To assess clinical applicability, SSOD_1 was compared with one senior ophthalmologist (SO) and three junior ophthalmologists (JOs) using identical clinical metrics on 127 single-lesion and 15 multi-lesion images, with detailed results shown in Table 7.

For single-lesion images, SSOD_1 achieved a total clinical score of 2.602/3—below the SO (2.992/3) and JOs (2.899/3)—yet still sufficient for disease screening. In multi-lesion evaluation, SSOD_1 performed comparably to the JOs (2.088/3 vs. 2.160/3) and, while lower than the SO (2.578/3), demonstrated adequate capability in complex scenarios. These findings suggest that, although SSOD_1 does not fully reach expert-level performance on single lesions, its multi-lesion detection approaches junior ophthalmologist-level accuracy, supporting its potential as a reliable clinical decision-support tool, particularly in settings with co-existing pathologies.

**Table 7:** Comparative evaluation of SSOD_1 and ophthalmologists on diagnostic accuracy, comprehensiveness, and location precision

Subject	Single-lesion (*N* = 127)					Multi-lesion (*N* = 15)			
	Accuracy	Comprehensiveness	Location	Total		Accuracy	Comprehensiveness	Location	Total
SSOD_1	0.906	0.850	0.846	2.602		1.000	0.544	0.544	2.088
SO	1.000	1.000	0.992	2.992		0.800	0.889	0.889	2.578
JO_1	0.945	0.976	0.969	2.890		0.600	0.778	0.778	2.156
JO_2	0.937	0.961	0.953	2.851		0.600	0.878	0.845	2.323
JO_3	1.000	0.992	0.965	2.957		0.600	0.701	0.701	2.002

## Discussion

Ocular anterior segment diseases are a major cause of visual impairment globally, profoundly affecting patients’ quality of life. Within the 3PM framework, timely and sensitive identification of these conditions is critical, particularly in complex clinical scenarios where multiple diseases may coexist. Early and interpretable detection systems can guide clinicians in tailoring individualized treatment strategies, preventing irreversible vision loss.

To address this clinical need, we developed a SSOD framework covering 12 common anterior segment diseases and their potential coexisting lesions. The system provides not only disease classification but also fine-grained lesion localization, offering interpretable outputs that enhance clinical transparency and actionable decision-making. In automated evaluation, the SSOD achieved overall mAP of 0.729 for single-lesion detection and 0.538 for multi-lesion detection. For clinically critical conditions, the model demonstrated robust performance with mAPs of 0.785 (single-lesion) and 0.705 (multi-lesion). Notably, when assessed at a screening-oriented operating point (confidence = 0.3), SSOD maintained a favorable balance between sensitivity and precision. These results indicate that SSOD can serve as an efficient and reliable tool for early anterior segment disease detection, supporting both preventive interventions and personalized treatment strategies.

From an algorithmic perspective, SSOD is tailored to the complexities of anterior segment screening. The semi-supervised architecture allows the system to achieve accurate predictions with minimal expert annotation, supporting rapid, scalable, and cost-effective large-scale screening programs. While classical fully supervised models such as Faster R-CNN [38] and YOLOv8 [37] provide strong baselines, their dependence on extensive manual labeling limits scalability in multi-disease, multi-lesion scenarios. Semi-supervised approaches such as Mean-Teacher [29] and Soft-Teacher [30] partially mitigate annotation burdens but often underperform in real-world settings due to class imbalance and open-set challenges. SSOD addresses these limitations through the CCE module, which dynamically balances disease representation, and the ODDFC, which incorporates previously unseen lesion patterns into pseudo-labeling. By enhancing predictive sensitivity, SSOD facilitates the shift from reactive to proactive, precision-oriented ophthalmic care, enabling individualized clinical decision-making in alignment with 3PM principles.

Rigorous automated evaluation and expert clinical validation confirmed the robustness of SSOD. Its performance was comparable to YOLOv8 in terms of mAP for both single- and multi-lesion settings (0.729 vs. 0.725 for single-lesion; 0.538 vs. 0.543 for multi-lesion). Similarly, under a screening-oriented operating point (confidence = 0.3), comparable performance was observed in terms of F1-score for both single- and multi-lesion settings (0.710 vs. 0.701 for single-lesion; 0.521 vs. 0.514 for multi-lesion). Notably, SSOD achieved superior recall and higher clinical scores (2.430/3 for single-lesion and 1.942/3 for multi-lesion detection), approaching the diagnostic performance of junior ophthalmologists in multi-lesion cases.

Previous studies have developed various models for anterior segment disease screening using slit-lamp images, but these approaches often focus on limited disease types [12–14] or lack interpretable lesion localization [16]. Most studies have focused on classification of a few selected diseases such as distinguishing keratitis from other corneal abnormalities [13], classifying fungal keratitis [39], differentiating among corneal ulcers and scars [14], or concentrated on conjunctivitis subtypes [12]. Ueno et al. expanded to nine categories including corneal and lens diseases, but excluded conjunctival disorders and did not provide lesion localization [17]. Li et al. introduced dense annotations with lesion-level localization, but their dataset was limited to six disease types, including pterygium, conjunctival oedema/hemorrhage/hyperaemia, keratitis, and cataract [15]. In contrast, SSOD covers 12 common anterior segment diseases, integrates multi-disease detection with interpretable lesion outputs, and demonstrates robust performance in multi-lesion scenarios that better reflect real-world clinical complexity. This combination of broad coverage, interpretability, and semi-supervised scalability represents a tangible step toward implementing 3PM-driven ophthalmic care.

## Conclusion and expert recommendations

We developed SSOD, a semi-supervised object detection system integrating CCE and OODFC modules, capable of detecting 12 common anterior segment diseases with interpretable lesion localization. The system is designed to operate effectively in complex clinical scenarios, accommodating both single-disease and multi-disease presentations. By enhancing early lesion recognition, supporting timely and targeted preventive interventions, and providing interpretable outputs for individualized treatment planning, SSOD embodies the core principles of predictive diagnostics, targeted prevention, and personalized management within the 3PM framework.

### Predictive diagnostics

The SSOD framework was specifically developed to improve early detection of anterior segment diseases, particularly in complex clinical scenarios with coexisting lesions. By providing interpretable lesion localization, the system allows clinicians to identify subtle abnormalities that might otherwise be missed. SSOD achieved an overall mAP of 0.729 for single-lesion detection and 0.538 for multi-lesion detection, along with F1-scores of 0.710 and 0.521, respectively, at a confidence threshold of 0.3. Notably, the model demonstrated superior performance for clinically critical conditions, with mAPs of 0.785 in the single-lesion setting and 0.705 in the multi-lesion setting. These results indicate that SSOD can reliably support early identification of vision-threatening lesions, enhancing predictive accuracy and facilitating timely risk stratification in accordance with 3PM principles.

### Targeted prevention

By reliably detecting early-stage abnormalities, SSOD provides actionable information to guide secondary prevention. Clinicians can use the system’s outputs to implement timely, disease-specific interventions that may prevent progression to irreversible vision loss. The framework’s efficiency and semi-supervised design make it suitable for large-scale screening, enabling rapid, cost-effective deployment in both primary care and resource-limited settings. The combination of high sensitivity and interpretability allows healthcare providers to focus preventive efforts where they are most needed, reducing the likelihood of missed diagnoses and optimizing patient outcomes.

### Personalized treatments

SSOD supports the detection of up to 12 common anterior segment diseases, significantly expanding coverage beyond previous AI systems limited to single or few conditions. The interpretable outputs provide precise lesion classification and localization, allowing clinicians to assess severity and spatial relationships, even when multiple lesions are present. This transparency enhances decision-making, enabling treatment plans to be tailored to each patient’s specific condition. The framework’s ability to handle multi-lesion presentations ensures that personalized interventions are informed by the full spectrum of observed pathology, promoting precision-oriented and patient-centered care in accordance with the 3PM paradigm.

### How does the presented study contribute to the paradigm shift from reactive to 3PM medicine and go beyond the state of the art?

This study advances the transition from reactive ophthalmic care to a 3PM framework by introducing SSOD, a semi-supervised object detection system tailored for anterior segment disease screening. Unlike traditional approaches that often target single lesions or rely heavily on clinician interpretation, SSOD provides interpretable outputs for up to 12 common anterior segment diseases, supporting early identification of vision-threatening conditions even in complex multi-lesion scenarios. The system’s semi-supervised architecture, combined with the CCE and ODDFC modules, enhances predictive sensitivity, ensures robust detection across both common and rare lesions, and accommodates previously unseen abnormalities.

By enabling precise lesion localization and multi-lesion analysis, SSOD supports targeted secondary prevention, guiding clinicians to implement timely, patient-specific interventions that may reduce the risk of irreversible vision loss. The framework also facilitates personalized treatment planning, allowing therapeutic decisions to be tailored according to lesion type, severity, and spatial distribution. Performance metrics, including overall mAP of 0.729 for single-lesion and 0.538 for multi-lesion detection, and high mAP for clinically critical conditions (0.785 for single-lesion; 0.705 for multi-lesion), demonstrate its reliability and clinical relevance.

Overall, SSOD operationalizes 3PM principles in a practical, scalable manner, surpassing previous AI models by simultaneously addressing multi-disease detection, interpretability, and clinical applicability. It transforms ophthalmic care from reactive, symptom-driven interventions to proactive, data-driven management, offering a concrete tool for predictive diagnostics, preventive strategies, and personalized treatments, particularly in primary and resource-limited settings.

### Limitations and outlook in the context of 3PM

Despite these strengths, SSOD has certain limitations. Its performance in multi-lesion scenarios, while adequate, is weaker than in single-lesion cases, requiring further improvement. Additionally, although the model excels in detecting clinically significant diseases, including cataract, lens dislocation, keratitis, corneal/conjunctival tumor and pterygium, its accuracy for specific conditions like corneal scarring and subconjunctival hemorrhage remains relatively lower. Furthermore, though included as many as 12 common lesions that may occur in clinical practice, other ocular anterior segment diseases not listed in the study can be missed by the system. Lastly, the model has yet to be externally validated across diverse imaging devices and patient demographics, which is essential for ensuring generalizability in real-world 3PM-oriented practice.

Looking forward, SSOD represents a meaningful step toward scalable and interpretable anterior segment screening. To further align with 3PM principles, future efforts should focus on: (1) expanding disease coverage and incorporating multi-source datasets to improve diagnostic comprehensiveness and equity; (2) integrating SSOD into intelligent clinical platforms, including large language models [40–44] and smartphone-based applications [17, 45–48], to support continuous monitoring, predictive triage, and personalized preventive care, fully realizing the 3PM-guided approach in ophthalmology.

## Data Availability

Due to the privacy of patients, the data related to patients cannot be available for public access, but are available from the corresponding authors on reasonable request approved by the human research ethics committee of the Second Affiliated Hospital of Zhejiang University.
